# Association of Serum Hepcidin With Preeclampsia: A Systematic Review and Meta-Analysis

**DOI:** 10.7759/cureus.26699

**Published:** 2022-07-09

**Authors:** Arkapal Bandyopadhyay, Farhad Ahamed, Sarika Palepu, Tandra Ghosh, Vikas Yadav

**Affiliations:** 1 Clinical Pharmacology, Netaji Subas Medical College, Patna, IND; 2 Community Medicine and Family Medicine, All India Institute of Medical Sciences, Kalyani, Kalyani, IND; 3 Physiology, All India Institute of Medical Sciences, Kalyani, Kalyani, IND; 4 Community and Family Medicine, National Institute for Research in Environmental Health, Bhopal, IND

**Keywords:** pre-eclampsia, ferritin, transferrin, hepcidin, iron parameters, pregnancy

## Abstract

The objective of the present systematic review and meta-analysis was to compare the levels of serum hepcidin in women who developed pre-eclampsia with those who did not. The databases PubMed, Embase, Scopus, Cochrane, and references of retrieved articles published till September 2020 were searched with no language restriction. Mean differences in iron regulating protein (hepcidin) were compared using a random-effects model based on the level of heterogeneity. A total of 760 individuals were included in the analysis from seven studies. The pooled estimate showed that mean hepcidin levels were significantly higher in women who developed pre-eclampsia [0.3 ng/ml, 95% confidence interval (CI): 0.01-0.59, p=0.003] as compared to women who did not develop pre-eclampsia. Further research can be done to assess the levels of various iron parameters in different trimesters of pregnancy and their association with pre-eclampsia.

## Introduction and background

Pre-eclampsia is a condition characterised by the development of vascular dysfunction leading to hypertension (systolic blood pressure ≥140 mm of Hg or diastolic blood pressure ≥90 mm of Hg) and proteinuria (>300 mg/24-hour urine), usually in the third trimester (after the 20th week) of pregnancy. Globally, around 5% of pregnant women suffer from pre-eclampsia, contributing to around 14% of total mortalities associated with pregnancy [1]. Similarly, in India, about 5% of primigravida suffer from pre-eclampsia, contributing to approximately 10% of total mortalities during pregnancy [2]. The primary pathophysiology of pre-eclampsia is suspected to be excessive vasospasm of uterine spiral arteries with anomalous placentation. Excessive vasospasm initiates a vicious cycle of ischemia and ischemic reperfusion injury, leading to inflammation and more vasospasm as a consequence [3].

Risk factors for pre-eclampsia are not completely understood yet. Apart from genetic and immunological factors, several other factors like infections (bacterial, viral, or protozoal), inflammation, and oxidative stress have been attributed to play a significant role in the pathogenesis of pre-eclampsia [4]. Although the precise role is still not clear, multiple studies have reported altered iron homeostasis as an important causative factor of pre-eclampsia. Several studies have also found that there is increased serum iron in pre-eclampsia and raised the possibility of a deleterious effect of excess iron as a result of blanket iron supplementation given during pregnancy [5,6].

Hepcidin, the master regulator in systemic iron homeostasis, reduces the availability of iron by decreasing intestinal iron absorption. It also blocks iron release from storage cells by down-regulation of ferroportin, the main iron exporter in mammalian cells. When the body's iron is depleted, hepcidin expression decreases and iron supply to plasma increases and vice-versa [7]. It has been found that in normal pregnancy, hepcidin levels decrease as pregnancy progresses. The lowest hepcidin level is usually observed during the third trimester of pregnancy, as maximum iron transfer from mother to foetus occurs during this time [8]. But, a complex picture has been observed in pre-eclampsia in terms of hepcidin concentration and serum iron parameters. Though the findings are not consistent, hepcidin has been found to be raised along with other iron parameters in pre-eclampsia even in the advanced stages of pregnancy [9]. To the best of our knowledge, there is no published systematic review and meta-analysis collating the evidence between hepcidin levels and pre-eclampsia.

The objective of this meta-analysis was to review the levels of serum hepcidin in pregnant women to understand their probable relationship with pre-eclampsia. The authors have also reviewed the levels of other iron parameters like transferrin saturation percentage and ferritin in the included studies.

## Review

Methodology

Search Strategy

This systematic review and meta-analysis were conducted according to the Preferred Reporting Items for Systematic Review and Meta-Analysis (PRISMA) guidelines [10]. The protocol has been registered on PROSPERO (Reference number - CRD42020200805).

A comprehensive, systematic literature search was conducted till September 2020 in PubMed/Medline, Scopus, and Embase databases. Separate search strategies were developed for these databases, consisting of a combination of free text words, words in titles/abstracts, and Medical Subject Headings (MeSH) for participants, study design, and study outcomes, and then combined by using the Boolean operator "AND" (Table 1). We placed no language or publication restrictions. We screened reference lists of identified studies and published reviews for additional studies. A search was also conducted in Cochrane to retrieve any additional studies.

**Table 1:** Search strategy Note: We searched for all three themes together using the Boolean operator ‘AND’ We used the lemmatisation, stemming and explode function for study population, exposure of interest and outcome of interest.

Criteria	Search terms
Study population	Pregnant* OR pregnancy OR antenatal OR prenatal OR “pregnancy complication” OR “pregnancy abnormality”
Terms of exposure	“Hepcidin” OR hepcidin [MeSH] OR “pro-hepcidin” OR “iron regulator” OR “membrane transport protein” OR “iron metabolism”
Terms of outcome	“Pre-eclampsia” OR “pregnancy-induced hypertension” OR “hypertension in pregnancy” OR “gestational hypertension” OR pre-eclampsia [MeSH] OR eclampsia

Study Selection Criteria

Inclusion criteria: The inclusion criteria were developed using the PECOS (Population, Exposure, Comparator, Outcomes, and Study characteristics) framework as follows: (i) studies on pregnant women reporting mean serum hepcidin levels and occurrence of pre-eclampsia; (ii) observational studies like cohort, case-control, cross-sectional studies and randomised controlled trials.

Exclusion criteria: (i) Studies without data on the control group (pregnant women without pre-eclampsia); (ii) individual case reports, case series, opinions, and review articles; (iii) studies with mean/median hepcidin levels and any pregnancy complication, but without specific details about hepcidin levels in patients with pre-eclampsia.

Literature Review and Data Extraction

AB and SP independently reviewed abstracts and obtained full texts of the articles for more details. VY and TG independently extracted and tabulated data from selected full texts. AB, SP, and FA reviewed the data according to inclusion and exclusion criteria, and any discrepancy was discussed and conclusions were drawn.

AB and SP independently performed complete data extraction of full texts to be included in the study. Study details, population characteristics, sample size, and hepcidin levels were tabulated. Details of other potential confounders, including age, parity, and trimester of pregnancy, were also recorded. Any disagreement in the data extraction was resolved by consensus and agreement was achieved by discussion with FA, VY, and TG.

**Figure 1:**
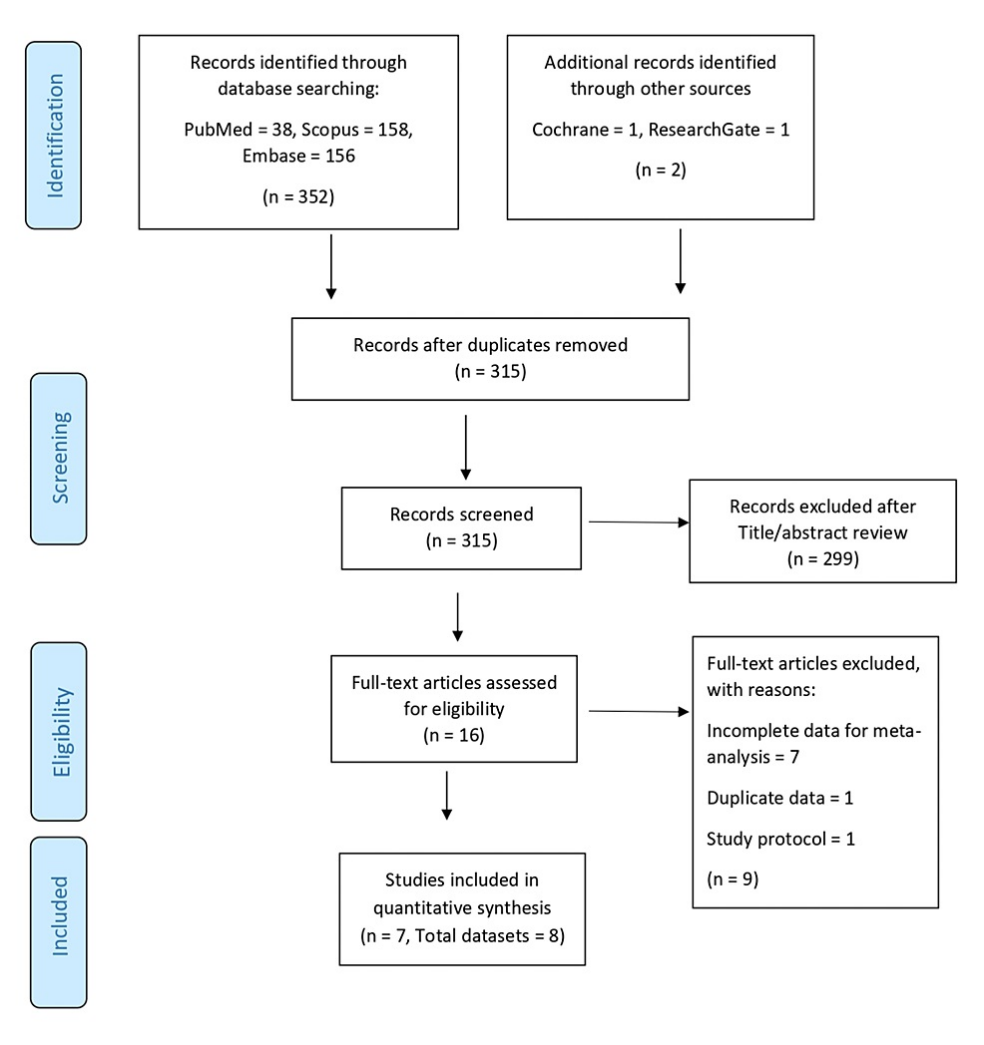
PRISMA flowchart of study selection process

The full-text copies of all selected articles were also evaluated for quality assessment. The study selection process is presented using the PRISMA flow chart (Figure 1).

Quality Assessment of Individual Studies

AB and FA independently assessed and scored the quality of observational studies included in this systematic review in accordance with the Newcastle-Ottawa Scale (NOS) [11]. Inconsistencies were resolved after discussion with SP, VY, and TG.

Data Synthesis and Analysis

In all the included studies, we estimated mean hepcidin levels in two groups of pregnant women-those who developed pre-eclampsia and those who did not. Data on mean/median hepcidin levels were converted to uniform units (ng/ml) from all the included studies. Standard deviation was calculated in studies that had median and inter-quartile ranges (SD = inter-quartile range/1.35). Heterogeneity between studies was examined using Cochran’s Q test and quantified using I² statistic. The pooled mean difference in hepcidin levels was calculated using a random-effects model, as the heterogeneity in the included studies was high (68%).

In the included studies, we also estimated mean differences in transferrin saturation percentage and levels of ferritin (ng/ml) between two groups using a random-effects model. Among the included studies, transferrin saturation percentage (if not available) was calculated using the formula "Saturation = (Serum iron concentration/Total Iron Binding Capacity) * 100." All analyses were done in STATA 17 (StataCorp LLC, TX) and Revman software.

Publication Bias

Publication bias was assessed by visual inspection of funnel plots and by evaluating the symmetry of the distribution.

Results

A total of 354 studies were obtained using the search strategy as described above (PubMed/Medline - 38, Embase - 156, Scopus - 158, and other sources - 2). After removing duplicates, a total of 316 studies were assessed for inclusion in the meta-analysis. Of them, 299 studies were excluded by reviewing the titles and abstracts as they were not relevant to the study question. The full texts of 17 studies were reviewed, and 7 were included in the present meta-analysis. The details of the articles reviewed are presented in the PRISMA chart (Figure 1).

Among the included studies, four were case-control, three were cross-sectional, and one was a prospective controlled study. In the included studies, 280 pregnant females developed pre-eclampsia and 480 pregnant women did not develop pre-eclampsia. The mean age of the pregnant women varied widely, from 18 to 40 years in these studies. The majority of the pregnant women were in the third trimester of pregnancy. Details of the included studies [12-18] are presented in Table 2.

**Table 2:** Characteristics of studies included in meta-analysis ^1^Pregnant females with normal fetal growth; ^2^Pregnant females with Intra-uterine growth retardation *N: sample size, NA: not available, GA: gestational age, PE: pre-eclampsia, KFT: kidney function tests, LFT: liver function tests Note: A study by Cardaropoli et al. [15] had two different study designs and two groups of study participants within the same study. Hence, this study was taken as 2 units and a total of 8 studies were included for meta-analysis.

Sl No	Author (study year)	Study characteristics	Study participants parameters		Other outcomes assessed
			Pregnancy without pre-eclampsia	Pregnancy with pre-eclampsia	
1	Toldi et al. [12]	Duration - NA, Budapest, Hungary, Cross sectional study	N = 37	N = 30	Iron homeostasis, interleukin-6 (IL-6), complete blood cell counts
			Median age - 30 years	Median age - 30 years	
			GA - 36 weeks	GA - 36.5 weeks	
			Parity - NA	Parity - NA	
			Hepcidin levels - 3.74 (0.73–8.14) ng/ml	Hepcidin levels - 5.68 (0.72–9.25) ng/ml	
2	Duvan et al. [13]	Duration - February 2010 to January 2013, Turkey, Case control study	N = 37	N = 30	KFT, LFT, iron markers, inflammatory markers
			Mean age - 30.2 ± 4.9 years	Mean age - 28.4 ± 5.4 years	
			GA - 39.3 weeks	GA - 35.2 weeks	
			Parity - NA	Parity - NA	
			Pro-hepcidin levels - 71.9 ± 22.1 ng/ml	Pro-hepcidin levels - 69.4 ± 19.7 ng/ml	
3	Muhsin et al. [14]	Duration - May to August 2013, Abu Dhabi, Case control study	N = 20	N = 20	Iron parameters
			Mean age - 28.3±6.3years	Mean age - 28.5±4.9 years	
			GA - Third trimester	GA - third trimester	
			Parity - NA	Parity - NA	
			Hepcidin levels - 556 ± 218 pg/ml	Hepcidin levels - 797 ± 249 pg/ml	
4	Cardaropoli et al. [15]^1^	Duration - October 2008 to August 2010, Turin, Italy, Cross sectional study	N = 60	N = 45 (20^1^, 25^2^)	PE risk factors, smoking status, expression of hepcidin gene on placenta, neonatal birth weight
			Mean age - 31.6 ± 4.6 years	Mean age - 34.2 ± 4.0^1^, 35.6 ± 4.7 years	
			GA - 33.7 weeks	GA - 33.3^1^, 31.3 weeks^2^	
			Nulliparous - 38	Nulliparous - 17^1^, 22^2^	
			Hepcidin levels - 48.50 ng/mL (41.28–62.63)	Hepcidin levels - 46.30 ng/mL (34.48–61.20)	
5	Cardaropoli et al. [15]^2^	Duration - October 2008 to August 2010, Turin, Italy, case control study	N = 228	N = 57 (47^1^,10^2^)	PE risk factors, smoking status, expression of hepcidin gene on placenta, neonatal birth weight
			Mean age - 31.4 ± 4.3 years	Mean age - 32.5±5.5^1^, 32.3± 5.1^2 ^years	
			GA - 13.5 weeks	GA - 13.8^1^, 14.6^2 ^weeks	
			Nulliparous - 127	Nulliparous - 33^1^, 7^2^	
			Hepcidin levels - 43.60 (33.88–55.67) ng/mL	Hepcidin levels - 50.56 (40.19–64.09) ng/mL	
6	Brunacci et al. [16]	Duration - 2010 to 2012, Sao Paulo, Brazil, Case-control study	N = 18	N = 18	Iron intake, haematological indices, iron status, LFT, inflammatory markers
			Mean age - 27 years	Mean age - 22 years	
			GA - 31.5 weeks	GA - 31.5 weeks	
			Nulliparous - 7	Nulliparous - 12	
			Hepcidin levels - 51.46 (47.73–59.81) ng/ml	Hepcidin levels - 46.52 (39.92–51.66) ng/ml	
7	Tapan et al. [17]	Duration - December 2015 – October 2017, Varanasi, India Case control study	N = 40	N = 40	Transferrin
			Mean age - 33.85 ± 4.63	Mean age - 30.7 ± 3.03	
			GA - 33.13 weeks	GA - 31.6 weeks	
			Parity - NA	Parity - NA	
			Hepcidin levels - 3.560 ± 2.48 ng/ml	Hepcidin levels - 5.773 ± 3.442 ng/ml	
8	Nila et al. [18]	Duration - NA, Tamil Nadu, India, Cross-sectional study	N = 40	N = 40	Iron homeostasis, oxidative stress, endothelial dysfunction
			Mean age - 18–40 years	Mean age - 18–40 years	
			GA - 34±4 weeks	GA - 34±4 weeks	
			Parity - NA	Parity- NA	
			Hepcidin levels - 684 (595–684) pg/ml	Hepcidin levels - 558 (425–610) pg/ml	

All the studies were found to be of good quality on assessment with NOS for case-control studies and modified NOS for cross-sectional studies (Table 3).

**Table 3:** Quality assessment of included studies *^,^**^,^***NOS uses predefined criteria and awards stars as scoring system for each study

Case-control studies: New Castle Ottawa Scale				
Study	Selection	Comparability	Exposure	Score
Muhsin et al. [14]	***	**	**	7
Cardaropoli et al. [15]	***	**	***	8
Brunacci et al. [16]	***	**	**	7
Tapan K et al. [17]	***	**	**	7
Duvan et al. [13]	***	**	**	7
Cross-sectional studies: Modified New Castle Ottawa Scale				
Study	Selection	Comparability	Outcome	Scoring
Toldi et al. [12]	*	**	**	5
Cardaropoli et al. [15]	*	**	**	5
Nila et al. [18]	*	**	**	5

**Figure 2:**
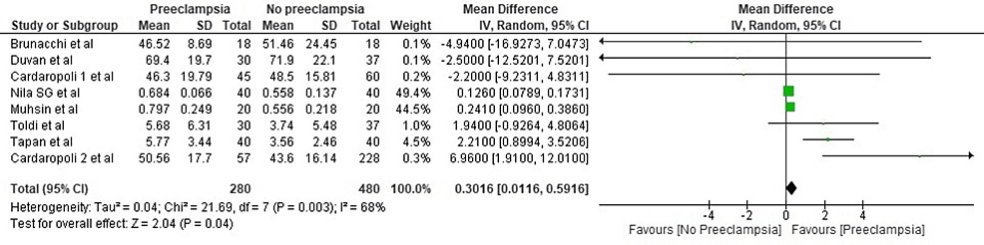
Forest plot of studies estimating serum mean hepcidin levels Sources: Toldi et al. [12], Duvan et al. [13], Muhsin et al. [14], Cardaropoli et al. [15], Brunacci et al. [16], Kumar et al. [17], Nila et al. [18]

The pooled estimate by the random-effects model showed that serum mean hepcidin levels were higher in patients who developed pre-eclampsia by 0.30 ng/ml. The mean difference was statistically significant (95% CI: 0.01-0.59, p=0.003) (Figure 2). The studies included had high heterogeneity, with an I2 value of 68%.

**Figure 3:**
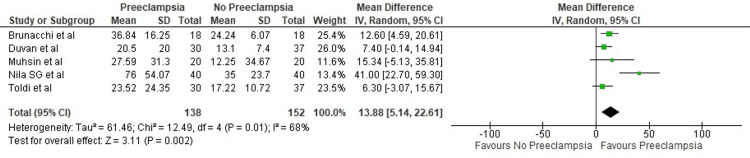
Forest plot of studies estimating transferrin saturation percentage Sources: Toldi et al. [12], Duvan et al. [13], Muhsin et al. [14], Brunacci et al. [16], Nila et al. [18]

Only five studies reported transferrin saturation and were included for analysis. It was seen that transferrin saturation was significantly higher in pregnant females who developed pre-eclampsia (13.88%; 95% CI: 5.14-22.61, p=0.01) (Figure 3).

**Figure 4:**
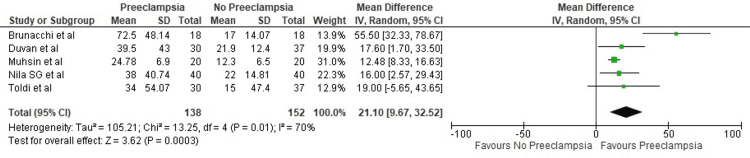
Forest plot of studies estimating ferritin levels Sources: Toldi et al. [12], Duvan et al. [13], Muhsin et al. [14], Brunacci et al. [16], Nila et al. [18]

It was seen that mean ferritin levels were significantly higher in women who developed pre-eclampsia (21.1 ng/ml; 95% CI: 9.67-32.52, p = 0.01) (Figure 4).

**Figure 5:**
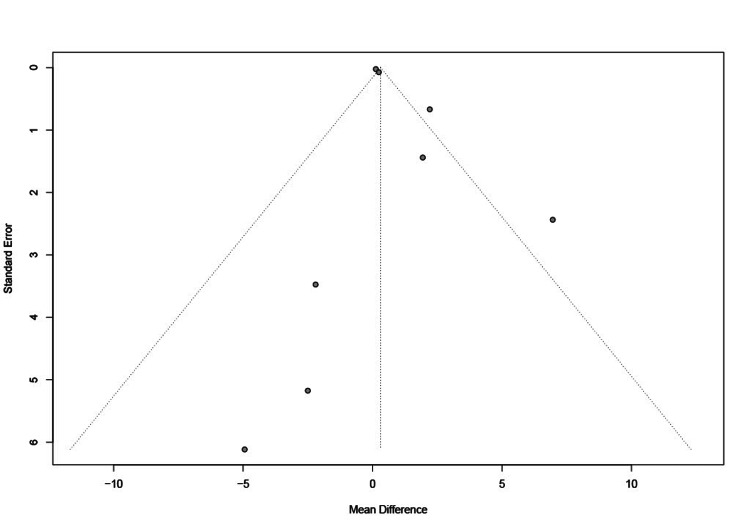
Funnel plot of included studies

The approximately symmetric distribution of funnel graphs ruled out the possibility of publication bias (Figure 5).

Discussion

Pre-eclampsia has been reported to significantly affect the mortality and morbidity of the mother and fetus. High blood pressure, proteinuria, or ultrasonography changes in uterine arterial flow after the 20th week of pregnancy have remained the main modalities of clinically diagnosing pre-eclampsia.

Early identification and risk assessment are prudent approaches to decrease feto-maternal morbidities. A biomarker that can be evaluated and followed up for early identification of the risk of developing pre-eclampsia is important for early prevention and management. Several studies have reported that serum hepcidin levels may have a potential association with the early diagnosis of pre-eclampsia. However, there are a few contradictory results. In the present study, we found that serum hepcidin, serum ferritin, and transferrin saturation percentage levels were higher in women with pre-eclampsia in comparison to women without pre-eclampsia.

Serum Hepcidin and Pre-Eclampsia

Hepcidin is the main molecule in the regulation of iron homeostasis in the body, and its level correlates with iron demand and iron availability. Hepcidin levels also correlate with inflammation, as it has been found that interleukin-6 (IL-6) induces hepcidin expression [12,18]. In addition, hepcidin levels also increase in infection with Helicobacter pylori and hepatitis C [19]. It has been demonstrated that specific antibodies against H. pylori can cross-react with placental tissue and result in poor placentation as seen in pre-eclampsia [20].

Although primarily hepcidin is produced by hepatic tissues, other tissues like placental trophoblasts also play a significant role in hepcidin production, though a complete understanding of this pathway is still lacking [15]. In normal pregnancy, maternal hepcidin expression is gradually suppressed during the second and third trimesters to increase the iron availability in the placenta to meet the high demand [21]. On the contrary, our results showed that hepcidin levels increased during the third trimester in women with pre-eclampsia. We have found that the mean difference in hepcidin levels in pre-eclampsia and normal pregnancy was 0.30 ng/ml (95% CI: 0.01-0.59).

This high level of serum hepcidin reflects multiple causative factors associated with pre-eclampsia. There is a possibility that excess labile iron absorbed through supplementation or generated by red cell damage due to ongoing inflammation/infection or oxidative injury may induce increased hepcidin expression in pre-eclampsia as a protective response to combat iron-mediated cytotoxicity [7,18]. Hepcidin up-regulation may also be due to local defense mechanisms against infection, e.g., H. pylori, causing retention of iron inside cells and decreasing the availability of essential iron to the pathogens [22]. But we could not comment on the aetiology of hepcidin up-regulation (due to infection or iron-mediated cytotoxicity) as all the studies except one [15] in the meta-analysis have analysed the level of hepcidin during the last trimester. The study by Cardaropoli et al. [15] showed that an increase in hepcidin level occurs as early as in the 13th week of gestation in pregnant females who develop pre-eclampsia. These findings give new hope to use hepcidin as a biomarker for the identification of at-risk mothers, but larger prospective studies are needed to confirm the same.

Serum Transferrin Saturation Percentage and Ferritin in Pre-Eclampsia

We observed that there was a significant difference in mean transferrin saturation percentage (13.88%, 95% CI: 5.14-22.61) and ferritin level (21.1 ng/ml, 95%CI: 9.67-32.52) with higher levels in pregnant females who developed pre-eclampsia.

As transferrin saturation is negatively associated with the iron demand in the body, increased transferrin saturation rules out iron deficiency in pre-eclampsia. Serum ferritin usually reflects the storage form of iron in the body [23]. Ferritin is also associated with inflammation in the body. An increased level of serum ferritin in pre-eclampsia may reflect the associated inflammation and confirm adequate storage of iron. In addition, an increase in serum ferritin during pre-eclampsia may be a part of the protective mechanism of the body to limit the availability of free iron, which is responsible for oxidative stress.

Thus, a significantly increased level of body iron (high serum ferritin and high transferrin saturation), both labile and storage forms of iron, has been found in pre-eclampsia compared to those who did not develop pre-eclampsia. Increased hepcidin with increased ferritin and transferrin saturation are quite contradictory to each other in the perspective of iron homeostasis. Whether the increase in hepcidin level is independently related to early infection and inflammation or whether it is a protective response to iron overload/iron-mediated cytotoxicity in pre-eclampsia needs to be identified through further prospective research. Similarly, as all the included studies in this meta-analysis except the study by Mushin et al. [14] reported a history of 30-60 mg/day of iron supplementation for pregnant women, we are unable to comment on the independent role of serum iron as a causative factor in pre-eclampsia.

The present meta-analysis is the pioneering research in evaluating the association between hepcidin and pre-eclampsia. As only a few normative data on hepcidin level is available, we are unable to comment on the reference range of hepcidin level in pregnancy with pre-eclampsia and without pre-eclampsia. But the present study definitely questions the policy of blanket supplementation of iron to all pregnant women. Continuing iron supplementation in pre-eclampsia may worsen the inflammatory condition by the generation of reactive oxygen species (Fenton reaction) [24], shifting the metabolic switch from aerobic glycolysis to oxidative phosphorylation [25], increasing transcription of hypoxia-inducible factor (HIF), leading to early-stage placental tissue damage [26], favouring pathogen replication, and impairing nitric oxide production needed for capillary endothelial cells [27]. Hence, cautious supplementation of iron needs to be weighed against the risk of excess supplementation in patients diagnosed with pre-eclampsia.

Limitations of the Study

The present study has a few limitations. As the majority of the studies have been carried out as cross-sectional in the third trimester, the aetiology of increased hepcidin (infective/inflammatory) cannot be commented upon. The analytical method for iron parameters and hepcidin estimation was not uniform across the studies. One of the included studies reported levels of pro-hepcidin, which could not be converted to hepcidin. Also, the level of normal serum hepcidin in pregnancy is still unknown. Further prospective studies are needed to establish the role and reference range of hepcidin in normal pregnancy as well as in pregnancy with pre-eclampsia.

## Conclusions

Pregnant women who developed pre-eclampsia have significantly higher levels of serum hepcidin. However, variations in the levels of these parameters over different trimesters of pregnancy are still unknown. Further prospective studies need to be conducted to monitor serum hepcidin and determine its role in assessing the early development of pre-eclampsia as it can be of great aid to the healthcare system. At the policy level, tailored supplementation of iron in patients diagnosed with pre-eclampsia can be implemented to enhance the well-being of mothers and newborns.
